# Factors affecting acceptance of at-birth point of care HIV testing among providers and parents in Kenya: A qualitative study

**DOI:** 10.1371/journal.pone.0225642

**Published:** 2019-11-22

**Authors:** Catherine Wexler, May Maloba, Melinda Brown, Natabhona Mabachi, Kathy Goggin, Brad Gautney, Beryne Odeny, Sarah Finocchario-Kessler

**Affiliations:** 1 Department of Family Medicine, University of Kansas Medical Center, Kansas City, Kansas, United States of America; 2 Global Health Innovations, Nairobi, Kenya; 3 Health Services and Outcomes Research, Children’s Mercy Kansas City, Kansas City, MO, United States of America; 4 School of Medicine, University of Missouri-Kansas City, Kansas City, MO, United States of America; 5 Global Health Innovations, Dallas, TX, United States of America; Sefako Makgatho Health Sciences University, SOUTH AFRICA

## Abstract

**Background:**

At-birth and point-of-care (POC) HIV testing are emerging strategies to streamline infant HIV diagnosis and expedite ART initiation for HIV-positive infants. The purpose of this qualitative study was to evaluate factors influencing the provision and acceptance of at-birth POC testing among both HIV care providers and parents of HIV-exposed infants in Kenya.

**Methods:**

We conducted semi-structured interviews with 26 HIV care providers and 35 parents of HIV-exposed infants (including 23 mothers, 6 fathers, and 3 mother-father pairs) at four study hospitals prior to POC implementation. An overview of best available evidence related to POC was presented to participants prior to each interview. Interviews probed about standard EID services, perceived benefits and risk of at-birth and POC testing, and suggested logistics of providing at-birth and POC. Interviews were audio recorded, translated (if necessary), and transcribed verbatim. Using the Transdisciplinary Model of Evidence Based Practice to guide analysis, transcripts were coded based on *a priori* themes related to environmental context, patient characteristics, and resources.

**Results:**

Most providers (24/26) and parents (30/35) held favorable attitudes towards at-birth POC testing. The potential for earlier results to improve infant care and reduce parental anxiety drove preferences for at-birth POC testing. Parents with unfavorable views towards at-birth POC testing preferred standard testing at 6 weeks so that mothers could heal after birth and have time to bond with their newborn before–possibly–learning that their child was HIV-positive. Providers identified lack of resources (shortage of staff, expertise, and space) as a barrier.

**Discussion:**

While overall acceptability of at-birth POC testing among HIV care providers and parents of HIV-exposed infants may facilitate uptake, barriers remain. Applying a task-shifting approach to implementation and ensuring providers receive training on at-birth POC testing may mitigate provider-related challenges. Comprehensive counseling throughout the antenatal and postpartum periods may mitigate patient-related challenges.

## Introduction

Early infant diagnosis of HIV (EID) is critical to identify HIV-positive infants and initiate them on antiretroviral therapy (ART). However, inefficiencies along the EID cascade of care (i.e., late presentation to care; long turnaround times for sample processing, mother notification, and ART initiation),[1–4] result in Kenyan infants not being initiated on ART until a median age of 17.1–25.1 weeks.[4, 5] This is well beyond the targeted ART initiation age of 12 weeks to reduce the risk of mortality and slow disease progression.[6]

Testing infants at birth with more efficient point of care [POC] HIV diagnostic technology is emerging as a strategy to streamline EID and minimize challenges with traditional central laboratory based HIV DNA PCR testing at 6-weeks of age. POC diagnostic technologies such as GeneXpert HIV-1 Qual[7] and Alere q HIV-1/2 Detect[8] are cartridge-based tests that can be processed at the hospital by trained clinical or laboratory staff. Results are available to the patient within 2 hours, offering the potential for same-day ART initiation for positive infants. Testing HIV-exposed infants at birth using POC technologies can result in more HIV-positive infants being identified and initiated on ART at younger ages than traditional testing strategies.[9–11] In Lesotho, HIV DNA PCR testing for HIV-exposed infants within 2 weeks of birth reduced infant age at ART initiation from 14.6 weeks to 6.1 weeks.[12] In a South African pilot study evaluating POC technologies, the median time from sample collection to HIV-positive infant ART initiation was reduced from 18 weeks with standard HIV DNA PCR testing to 0 days with POC.[13] Studies have found that POC implementation is feasible in health facility-based settings in Kenya,[14] South Africa,[10, 15, 16] Mozambique,[17] and Tanzania[18] and is acceptable to providers.[16] Based on this evidence, Kenya and other countries are incorporating at-birth and POC testing into national plans for EID.[19–21]

**Fig 1:**
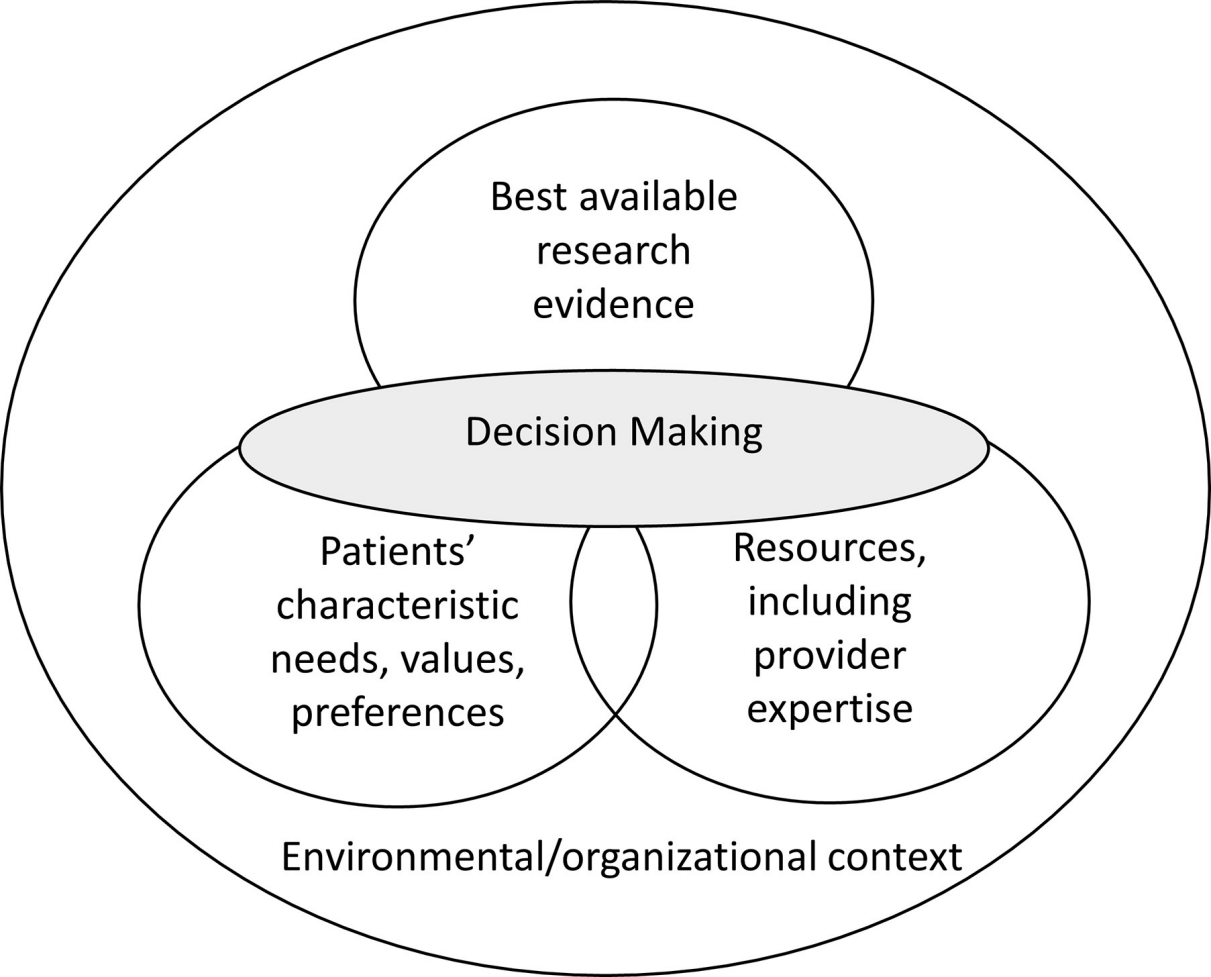
Transdisciplinary model of evidence based practice. Using the Transdisciplinary Model of Evidence Based Practice [28] to guide analysis, the purpose of this qualitative study was to evaluate factors that may affect the decision to provide or accept at-birth POC testing among both HIV care providers and HIV-positive parents in Kenya.

While at-birth and POC strategies to expedite infant diagnosis and treatment hold great promise, dissemination of prevention of mother to child transmission (PMTCT) innovations in sub-Saharan Africa, including Kenya, has historically been slow.[22–26] This suboptimal dissemination of best practices has resulted in a growing emphasis on grounding policy recommendations in evidence and sound behavioral theory.[23] Evidence based practice (EBP) refers to a process of decision making that promotes using clear evidence and justification to decide a course of action, rather than more subjective measures like intuition, personal experience, and habit.[27] The Transdisciplinary Model of Evidence-Based Practice proposes that the decision to adopt interventions, such as birth POC testing, should be influenced by three factors: (1) the best available evidence regarding the efficacy of the intervention, (2) available resources, which includes provider expertise, knowledge, and attitudes, and (3) patients’ characteristics, needs, values, and preferences.[28] The model also recognizes that external to these three factors, environmental and organizational variables related to the community, clinic and health system environment influence adoption [Fig 1]. Often these factors do not align (e.g., patient preferences are at odds with the best available evidence or resources; resources are insufficient to accommodate what the evidence suggests) and it is up to providers to reconcile them and present patients with adequate information to make an informed decision,[28] highlighting the complexity of decision-making in clinical care. Shared decision making, in which patients and providers share information and engage in discussion to collaboratively reach a decision, is a critical component of EBP. Without a participatory and open relationship between patients and providers, clinicians cannot adequately assess patient’s values and preferences to reach an EBP-informed decision.[29, 30]

## Methods

### Study design

This qualitative study was part of the formative component of a pilot study to assess the feasibility and effectiveness of GeneXpert and Alere q for at-birth POC HIV testing. The aim of the formative component was to assess factors that could facilitate or impede acceptance of POC testing and strategies to optimize implementation. At the time of the interviews, POC machines had not yet been introduced to the study hospitals for testing at any time point and no training for POC implementation or study procedures had occurred.

### Study setting and participants

This study was conducted at 4 Kenyan government hospitals. The hospitals were located in coastal (n = 1), central (n = 1), and western (n = 2) Kenya and were of varying resource levels (district hospital (n = 2), county referral hospital (n = 1) and a provincial referral hospital (n = 1)). Annual EID volumes in 2017 ranged from 159 and 704 and the percentage of HIV-exposed infants testing positive at 6 weeks ranged from 1.3% to 4.1%.[31]

At the time of the study, Kenyan National EID guidelines (2016) recommended HIV DNA PCR testing for all HIV-exposed infants at birth, 6 weeks, 6 months, and 12 months, with a final antibody test at 18 months.[32] If at any time an infant tested positive, guidelines recommended immediate initiation of ART.[32] Despite at-birth testing being included in the guidelines, implementation was delayed until results from national piloting could be evaluated, thus neither at-birth testing nor POC testing (at any testing time point) was being implemented nationally at the time of the study.

We conducted semi-structured interviews with 26 key PMTCT/EID providers at the 4 study hospitals and n = 35 parents of HIV-exposed infants (including 23 mothers, 6 fathers, and 3 mother-father couple pairs) who accessed EID services at one of the hospitals in February and July of 2017. Provider participants were purposively selected by study staff to represent different departments (Laboratory, Maternity, and Maternal Child Health [MCH]) and different roles (clinical officer, nurse, lab manager, lab technician, mentor mother [HIV-positive mothers who have been through EID and provide a range of services including case finding and referral, defaulter tracing, case management, support group facilitation, health education, and support for enrollment, retention, and adherence], and midwife) involved in the provision of PMTCT and EID services. All relevant providers available at the hospital on the days the interviews were conducted were asked to participate; thus, the sample size for provider participants was limited by the number of relevant provider at each site. Parent participants were recruited by each hospital’s mentor mothers and were contacted by phone or in person to participate. Parent interviews were scheduled on clinic days when mothers were already to be at the hospital for their own or their infant’s care. We targeted an initial sample size 10 parent participants per site. After 10 participants, the research team reviewed field notes and preliminary findings during a debriefing session and assessed the level of saturation (i.e., no newly emergent themes, high coverage of a priori and emergent themes). At sites where saturation had not been achieved after 10 participants, additional interviews were conducted and discussed until consensus on saturation was reached. Due to a strike, which created logistical constraints related to scheduling parent participants, only two parents were available for interview at one of the hospitals. No participants refused to be interviewed. Hospital, provider, and parent details are outlined in Tables 1 and 2.

**Table 1:** Provider details.

Code	Provider Details
Country Referral Hospital	
Provider 1	Mentor mother
Provider 2	Nurse, MCH in-charge
Provider 3	Lab technician
Provider 4	Nurse-midwife, Maternity in-charge
Provider 5	MCH nurse, midwife
Provider 6	MCH nurse
District Hospital 1	
Provider 7	Nurse, MCH in-charge
Provider 8	Lab Manager
Provider 9	Maternity nurse
Provider 10	MCH nurse
Provider 11	Nurse-midwife, Maternity/MCH in-charge
Provider 12	MCH & Maternity Clinical officer
Provincial Referral Hospital	
Provider 13	MCH nurse
Provider 14	Nurse in charge
Provider 15	MCH nurse
Provider 16	Midwife
Provider 17	Mentor mother
Provider 18	Lab Technician
Provider 19	MCH nurse
District Hospital 2	
Provider 20	Lab Manager
Provider 21	Lab technician
Provider 22	MCH nurse
Provider 23	MCH Nurse
Provider 24	MCH Nurse in-charge
Provider 25	Maternity nurse
Provider 26	MCH Nurse

**Table 2:** Parent details.

Code	Parent Type	Disclosure Status^a^	Timing of Diagnosis^a^
County Referral Hospital			
Parent 1	Mother	Disclosed	KP
Parent 2	Mother	Unknown	Unknown
Parent 3	Mother	Disclosed	ND
Parent 4	Father	Disclosed	KP
Parent 5	Mother	Unknown	KP
Parent 6^b^ Parent 7	Couple	Disclosed	KP
Parent 8	Father	Disclosed	Unknown
Parent 9	Mother	Unknown	ND
Parent 10	Father	Disclosed	Unknown
Parent 11	Mother	Disclosed	KP
Parent 12	Mother	Disclosed	ND
District Hospital 1			
Parent 13	Mother	Unknown	ND
Parent 14	Mother	Unknown	KP
Parent 15	Mother	Unknown	Unknown
Parent 16	Father	Disclosed	Unknown
Parent 17	Father	Disclosed	Unknown
Parent 18	Mother	Unknown	Unknown
Parent 19	Mother	Unknown	Unknown
Parent 20	Mother	Unknown	Unknown
Parent 21	Mother	Unknown	KP
Parent 22	Mother	Unknown	ND
Provincial Referral Hospital			
Parent 23	Mother	Unknown	ND
Parent 24	Mother	Disclosed	KP
Parent 25	Mother	Disclosed	KP
Parent 26	Father	Disclosed	Unknown
Parent 27	Mother	Unknown	Unknown
Parent 28^b^ Parent 29	Couple	Disclosed	KP
Parent 30^b^ Parent 31	Couple	Disclosed	KP
Parent 32	Mother	Disclosed	Unknown
Parent 33	Mother	Disclosed	KP
District Hospital 2			
Parent 34	Mother	Disclosed	ND
Parent 35	Mother	Disclosed	ND

^a^parents’ disclosure status and timing of diagnosis were not directly assessed. These characteristics were deduced from answers to other questions, resulting in missing information for many parents.

^b^denotes the mother in a couple

KP = known positive prior to last pregnancy; ND = newly diagnosed at time of last pregnancy

Written informed consent was obtained from each participant prior to the interview and a remuneration of 500 Kenyan Shillings (approximately USD $5.00) was given in appreciation of participants’ time. All study protocols were approved by ethical review committees at the Kenya Medical Research Institute and the University of Kansas Medical Center.

### Overview of best available evidence

Prior to conducting e**a**ch interview, the interviewer introduced him/herself, explained the purpose of the study, gave each participant an information sheet outlining the best available research evidence for POC testing using the GeneXpert and Alere q machines, verbally described the process of collecting and processing samples, and discussed the sensitivity and specificity of the results. While some providers had experience using GeneXpert for tuberculosis diagnosis, this was the first time that all parents and most providers were learning of the use of POC machines for early infant diagnosis of HIV.

### Data collection and analysis

All interviews were conducted by on-site research coordinators or the study manager (MM, NM, MB) who received training in qualitative data collection methods. Interviewers had no prior contact with parent participants, but had previously interacted with some providers during the process of funding acquisition, IRB approval, and study start up. The data reported in this manuscript focus on interview question topics related to factors impacting acceptance and perceived benefits and barriers of at-birth POC testing. Interviews lasted approximately 45 minutes and were conducted in a private office at the study hospital. Provider interviews were conducted in English, audio recorded and transcribed verbatim. Parent interviews were conducted in the participant’s language of choice (English, Swahili, or local language [Luo]), audio recorded, translated, and transcribed.

Transcripts were coded independently by three study team members (CW, SFK, MB) in Excel [33, 34] based on *a priori* themes corresponding to the constructs of the Transdisciplinary Model of Evidence Based Practice [28]: environmental context, patient factors (characteristics, needs, preferences), resources (provider expertise, attitudes, constraints), and best available evidence. Coding discrepancies were addressed through group consensus, and the frequency and distribution of the final codes were noted.

## Results

**Fig 2:**
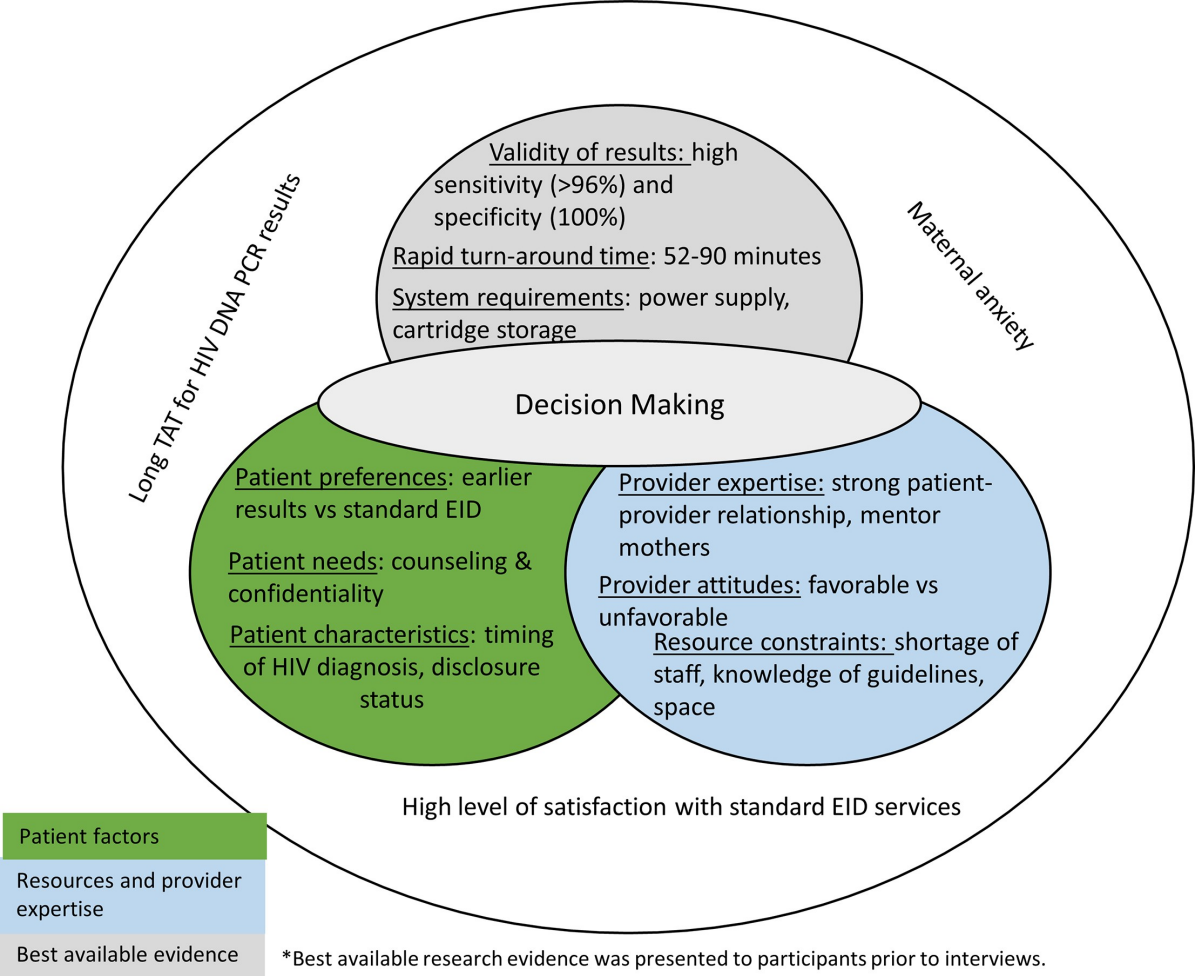
Factors impacting the provision and acceptance of at-birth POC testing.

We organize the results by first discussing the environmental context: parent and provider perspectives of standard EID services. Next, we present the patient, resource and provider-related factors affecting provider and patient uptake of at-birth POC testing. Under “best available research evidence”, we briefly discuss participants’ assessments of the evidence presented to them. Fig 2 shows the themes and topics discussed pertaining to each area of share decision making surrounding at-birth POC testing.

### Environmental context

Both parents and providers discussed how long turnaround times for infant HIV DNA PCR test results were a challenge with standard EID services, with results often taking more than a month to reach mothers:

“*The turnaround time used to be even two months*. *Then now you notify this mother another one week or two weeks*. *So that problem has been there”* (Provider 7).

During this long waiting time for infant results, most parents felt substantial anxiety, “*You cannot predict [the result]*. *So waiting for the results is terrifying*.” (Parent 31)

However, a few parents stated that they *“…didn’t have too many worries…they had told me that if I tend to the child as advised*, *the chances of getting sick would go down*. *So*, *I knew the child was safe*.*”* (Parent 2)

Despite the long turnaround times for HIV DNA PCR test results, most parents expressed satisfaction with their EID experience, including satisfaction with the quality and efficiency of services provided, comprehensiveness of counseling, and the attitudes of hospital staff:

*“And then the services down at the maternity are very good*. *The nurses are cooperative* … *When they [HIV+ mothers] come to the hospital*, *they are given priority*. *They don't have to take time in the hospital*. *They get what they want*, *the drugs*, *the counseling*, *part of it*.*”* (Parent 14)

### Patient factors

#### Patient preferences

Overall 30 of the 35 (85.7%) parents held a favorable view towards at-birth POC testing. Most parents expressed a preference for earlier results. Similarly, providers felt that parents would prefer and benefit from getting their infants’ results earlier. Both parents and providers agreed on two primary benefits that earlier results would afford to parents: it would reduce parental anxiety caused by long waiting periods and it would improve infant outcomes by allowing clinicians to provide result-based counseling and services earlier.

*“Testing for HIV at birth*, *I think it's a good idea*, *because it makes someone be relieved of that anxiety*. *At least you will want to know how is the child*, *is she positive or negative*?*”* (Parent 11)

*“When they test that early they will know what they will do*. *They will evaluate these that were tested and the outcomes of those results to pave a way forward*.*”* (Parent 23)

Several providers noted that mothers were often lost to follow up prior to the six week test. Providers felt that at-birth POC testing, allowing women to get their infant’s test and result on the same day, would reduce the occurrence of missed opportunities “…*because we can get those babies' status even before they go home*. *And some of our mothers don't come back for those post-natal clinics we give*. *So if we can do it during the delivery or just before they go home*, *it will help capture those babies early*.” (Provider 14) This was seen as especially relevant for parents who may have traveled far to reach the hospital because *“you don’t know where they come from*, *specifically* … *If she or he misses that test at that particular time*, *sometimes she will not have another opportunity to do that test*.” (Provider 4)

Parents and providers also noted that it could reduce the rates of loss to follow up by motivating continued engagement in care and adherence to PMTCT recommendations.

*“For me*, *I think it will make mothers do a lot of follow up [rather] than defaulting*. *Because if at birth*, *if I’m a seropositive mother and I find out immediately after birth—when I think the blood has mixed a bit—my baby turns out to be negative*, *it will motivate me to continue knowing if it will be continuously negative [at follow up testing]*.*”* (Parent 14)

While most parents expressed their own preference for earlier testing and same-day results offered by at-birth POC testing and providers agreed that this would be beneficial to parents, both sets of stakeholders offered insights on why parents may reject this method of testing, instead preferring standard infant testing at 6-weeks of age. Fear that an HIV-positive result at birth would preclude parent-child bonding and negatively impact infant care was expressed by several parents:

*“You can’t accept to raise the baby*, *and he has a problem ahead*. *So many might have bad thoughts*. *So it’s better at six weeks so people know you have a baby*, *and you have love for the child*.*”* (Parent 25)

Fear that at-birth POC testing could risk unintentional disclosure if “*someone has brought the mother to the hospital and this person doesn’t know the status of the mother then there is a risk disclosure happening*” (Parent 32) was expressed by some parents; however, more felt that “*disclosure may not happen since they wouldn’t know why blood is being drawn*.” (Parent 27). Similarly, providers felt that issues of stigma and disclosure would not pose significant challenges to at-birth POC testing.

Other stated reasons that parents may be resistant to birth POC testing were: (1) after delivery “*some period of time should be given to the mother so that [the mother] can heal*” (Parent 1) both physically and emotionally; (2) It may decrease EID retention “*because they will opt not to participate in EID since they get the results on the same day*” (Parent 27) and, thus, may forego follow up testing; (3) they “*think the baby will feel too much pain*” (Parent 32) if a sample is taken at birth; and (4) “*Some will be scared about the same day results especially those who have not been looking after their children well*.*”* (Parent 5)

#### Patient needs

Patients’ need for comprehensive maternal counseling, starting in pregnancy and continuing through delivery and postpartum was mentioned by nearly all parents and providers. Counseling was seen as necessary to support acceptance of at-birth POC testing, acceptance of their infant’s result, and appropriate infant care based on the result of a birth POC test.

*They should prepare them through having health talk…when they’re pregnant*. *They should know at the time they are four months pregnant*, *six*, *seven like that so they become prepared*. (Parent 16)

It need a lot of counseling…They will not be happy of the [positive] result. Maybe that one was your first child and, ‘Wow, I have a child and she also sick, what can I do? What will I do with this child?’ So they need just counseling. (Parent 22)

Some parents also discussed how social support provided by the parent’s family/friends, by a mentor mother or other provider, or by other clients at the hospital would facilitate parental acceptance of at-birth POC testing, “*The thing that motivated me to do this [receive EID care]*, *through the doctor's instructions*, *how they encourage people*. *If you come here*, *you find so many people*, *they also testing HIV positive*, *so that will* … *It encourages you*, *so that you see that you are not alone*.*”* (Parent 11)

#### Patient characteristics

Parents and providers discussed how the timing of maternal HIV diagnosis and maternal disclosure status could impact mothers’ acceptance of at-birth POC testing. More resistance was anticipated among mothers newly diagnosed as HIV-positive during their current pregnancy or delivery, compared with mothers who had known their HIV status for some time:

*“Unless this one is a mother who was tested*, *maybe*, *two*, *three days back*, *or was tested at delivery*, *or at maternity*. *Had never been on care*, *and fears the baby may be infected*. *It's the only person who may not want to receive the result*.” (Parent 14)

Similarly, mothers who had not disclosed their own HIV status to family and/or friends were seen as less likely to accept at-birth POC testing for their newborns because if “*she has not disclosed to the husband and testing the baby [at the time of delivery] would make the husband know the situation*.” (Parent 26)

Despite these qualitative comments from providers and patients that a new diagnosis or nondisclosure may impede acceptance of at-birth POC, review of individual participant data revealed that among the 20 parents with known timing of diagnosis, all newly diagnosed parents (8/8) held favorable views towards at-birth POC testing compared to only 66% (8/12) favorable views among parents who were previously diagnosed. Among those known to have disclosed their status, 73.7% (14/19) held favorable views towards at-birth POC testing, compared to 100% (16/16) with unknown disclosure status.

### Resources

#### Provider expertise

Both providers and parents discussed how parents have a high level of trust in providers and, thus, would be willing to follow provider recommendations for at-birth POC testing.

*“It depends on what the sisters say*, *and you follow their advice*.” (Parent 14)

*“Once they have heard themselves that professionals themselves are handling this [birth POC testing]*, *I don’t think it is going to cause any effect to them*. *Because they trust the clinician*.*”* (Provider 3)

*“Mentor mothers were very helpful since they prepare you from pregnancy that you baby will be tested so when the time reaches for the baby to be tested one is very confident*.*”* (Parent 33)

However, providers felt that their current knowledge of both the 2016 guidelines recommending at-birth testing and the use of the POC machines was inadequate to support this initiative. They felt that their lack of expertise in these areas would impact their ability to provide quality at-birth POC testing to clients because, “*If they are not trained*, *then it means you are not competent and you cannot do that work properly to the standard that is needed*.” (Provider 5) However, this barrier was seen as easily overcome with staff training.

“S*o majority of the healthcare workers are not aware of the new guidelines*, *so we need to sensitize these people*. *They need to be trained so that when they come across a mother*, *they are able to tackle that mother fully without referring to another section*.*”* (Provider 16)

#### Provider attitudes

Nearly all providers (24/26, 92.3%) held a favorable view towards at-birth POC testing. Like parents, providers’ enthusiasm for at-birth POC testing was driven by anticipated improvements in clinical management for HIV-positive infants:

“*The benefits are*, *we'll be able to attend to the child according to their immediate needs*. *If the result for this baby is positive*, *you will be able to put interventions immediately*. *Maybe you start the child on full ART*. *If this child is negative*, *the mother will be supported and maybe referred to the MCH for a test again in six weeks*. *So if it is beneficial to both the mother and the health worker*.” (Provider 15)

The two providers who held and unfavorable view of at-birth POC testing misunderstood the mechanism of testing and how it differed from rapid antibody tests. These providers were concerned with mothers and babies “mixing blood,” which, they feared, would cause a false positive POC result.

*“Previously we used to say that a baby born to a sero-exposed mother may have the*, *it’s called what*, *the*, *the antibodies of the mother in the blood and that can give a false positive result*. *So what has changed*?*”* (Provider 4)

#### Resource constraints

While enthusiasm facilitated provider willingness to accept at-birth POC testing, they feared that significant resource constraints would hinder their ability to successfully implement the intervention. Both patients and providers stated that “…*the patients are many*. *And the workers are few*.*”* (Parent 31) This shortage of adequately trained personnel was seen as the primary resource constraint to at-birth POC testing. Given their current staffing levels, most providers felt that the extra work required to provide maternal counseling and collect and process samples would result in provider burnout and/or inability to manage at-birth POC testing and other responsibilities.

*“This testing and counseling is also time consuming so it will limit your time on the other activities that you are supposed to do*. *So I think it [POC testing] will impact on the performance of other activities*.*”* (Provider 4)

Insufficient space in maternity was seen as additional resource constraint that would impact the provision and acceptance of at-birth POC testing because it may impede adequate confidentiality during counseling and testing:

*“I would want to have an office where that specific testing and counseling is done so that it is done within a room to maintain the privacy and confidentiality of the mothers… We don’t have [a room]*. *That is the challenge*.*”* (Provider 4)

### Participant assessment of best available evidence

Given the novelty of testing at birth and with POC HIV diagnostics for infants, providers were largely unaware of research evidence regarding at-birth POC testing. Prior to the interviews, research staff discussed the machines and gave providers a brief overview of current literature. In the absence of other evidence or personal experience using the machines, some trusted that “…*this thing*, *the machine*, *is of quality and can give great results*.*”* (Provider 5) However, others were more skeptical and questioned if the machine met their standards and insisted on creating their own evidence through machine validation tests, prior to full implementation:

*“We need to see if this equipment is validated—can give the same results as the other equipment*. *So once we validate*, *and we know the equipment is working again like other equipment*, *then we will not have the problem*. *But at first*, *we do have a concern about the validation*.*”* (Provider 3)

Similarly, a few parents were skeptical that such rapid results would be as reliable as the PCR results. These parents suggested, “*you cannot hasten the process of getting the result if that the is the normal time to wait since this is something that they have done research on and they know it should take this amount of time*.” (Parent 23)

## Discussion

Decision-making around the adoption of at-birth POC testing will be influenced by provider and parent preferences in the context of limited resources and limited provider experience with POC. The majority of parents and providers held favorable attitudes towards at-birth POC testing for HIV-exposed infants, stating that the earlier results provided by at-birth POC testing would reduce parental anxiety and improve infant care. This qualitative finding is supported by quantitative studies suggesting that POC testing can result in earlier ART initiation for HIV+ infants and improved viral suppression at 6-months.[10, 11, 13] Furthermore, both parents and providers felt that an initial negative result would motivate continued parental engagement in EID care, an important finding since routine testing through 18 months (or the cessation of breastfeeding) is still indicated by national guidelines to rule out intrapartum and breastfeeding transmission. Contradicting this qualitative finding, a study in South Africa found that infants tested negative at birth were significantly less likely to return for follow up care than infants not tested at birth.[11] It is unknown how an indeterminate or failed at-birth POC test may impact continued EID care. However, even with some disengagement after birth testing, HIV testing at both birth and at 4–6 weeks can still identify more HIV-infected infants than testing at 4–6 weeks alone because of high rates of infant loss to follow up between birth and 6-weeks.[9, 11] Future studies should evaluate barriers to repeat testing after initial birth testing faced by patients and health systems. In order to maximize the benefit of at-birth POC testing, it will be essential to address these barriers and comprehensively counsel parents on the need to return at later testing time points.

Despite favorable attitudes, significant structural barriers in terms of insufficient resources (insufficient staffing, expertise, and infrastructure) and limited evidence available to clinicians were identified. These findings are consistent with a Malawi study which found that although nurses had average knowledge and positive attitudes towards the evidence based practice in PMTCT, insufficient resources and difficulties accessing new information (through scientific journals and national/international guidelines) hampered efforts and caused low implementation of EBP.[35] If at-birth POC is to be successfully integrated into routine decision making, these challenges need to be overcome.

Shortage of clinical personnel was seen by providers as the primary constraint to providing at-birth POC testing. Indeed, the shortage of health workers in many African countries is recognized as a crisis[36] and has been often cited as a challenge to providing PMTCT/EID services in Kenya.[37, 38] In Kenya, estimates of provider-patient ratios range from 8–15 providers per 10,000 populations, significantly lower than the WHO recommended 25 per 10,000 population, resulting in shorter consultations for patients, clinician burnout, and can lead to slow adoption of clinical guidelines and suboptimal patient care.[36, 39, 40] Task shifting offers the potential for clinical responsibilities to be shifted from physicians to nurses and nurses to lay health workers[41, 42] and has been adopted by Kenyan national policy.[43] Distributing responsibilities between personnel in different roles will reduce the amount of additional work that any one cadre of provider will be tasked with and will maximize the chances of successful implementation.[14]

Evidence related to PMTCT and EID is a rapidly changing field, prompting frequent guideline updates.[44] In Africa, limited access to and comprehension of available evidence through medical journals, international recommendations, and other sources has been cited as a significant barrier to the uptake of EBP.[35, 45] This inaccessibility results in clinicians often relying on pre-service training, recommendations from colleagues, intuition, and in-service trainings, rather than comprehensive review of evidence.[45] In our study, providers recognized that lack of familiarity with the newest guidelines [32] and lack of knowledge regarding POC testing posed barriers to successful implementation and adoption. Providers suggested training and internally-conducted machine validation tests to bolster the limited evidence they had available. These solutions provide an easy way to quickly enhance provider expertise and knowledge of evidence regarding at-birth POC.

Given some parents’ resistance towards at-birth POC testing, providers will need to engage patients in shared decision-making early (during pregnancy) to ensure that an individual patient’s preference is understood and respected. As noted by both clinicians and patients in the current study, counseling parents on the potential benefits and risks associated with at-birth POC testing will be necessary to help patients make an informed decision. Counseling should be a continuous process, beginning early in pregnancy and continuing throughout the delivery and postpartum periods to support testing uptake and acceptance of results. The rate of facility delivery in pregnant women living with HIV in Kenya is high (97%)[46]. While this high rate of facility-based delivery could be leveraged to facilitate at-birth POC testing, care must be taken to ensure that parents understand alternatives to at-birth POC testing. As noted in this study and others,[47, 48] patient trust in healthcare workers is high, often leading patients to accept services without fully understanding them.[48, 49] Some studies have noted that Kenyan physicians have a directive communication style; often not assessing patient preferences, encouraging questions, or making all available options known to the patient.[47, 48] In response, patients may exert their decision-making power by dropping out of care, rather than refusing specific services.[48] Thus, in order to support continued engagement in PMTCT/EID care, providers should ensure that parents understand their options and help parents make a decision that respects their preferences and supports sustained engagement in care.

This study has at least three limitations, which should be noted. First, all data collection occurred prior to implementing at-birth POC testing and thus represent perceived facilitators and barriers, rather than realized benefits and barriers. Additional data from focus groups conducted throughout the pilot period will help to confirm if the perceived facilitators and barriers identified in this study hold up once the participants experience the process. Second, all parent participants were engaged in EID care for their HIV-exposed infant and were selected by mentor mothers who they have an ongoing relationship with, thus their views towards at-birth POC testing may not be representative of patients who are less engaged in care. This may account for the discrepancy we saw between the perceived impact that timing of diagnosis and disclosure would have on acceptance of at-birth POC testing. These two factors have been found to influence maternal and infant engagement in HIV care.[50–52] Among those already engaged in EID care, these factors may be less predictive of their acceptance of a certain intervention than in the general population. Third, we did not collect demographic data (participant age, infant age at time of interviews, etc) on participants at the time of the interviews; thus are unable to comment on how perceptions of standard at-birth POC may vary based on participant characteristics.

## Conclusion

Most HIV-care providers and parents of HIV-exposed infants held favorable views towards at-birth POC testing and felt that earlier results provided by at-birth POC testing would benefit parents and children by reducing parental anxiety and informing infant care. However, a few participants preferred standard testing so that mothers could heal after birth and had time to bond with their newborn before–possibly–learning that their child was HIV-positive. Counseling, starting early in pregnancy and continuing through delivery, and ensuring confidentiality will facilitate acceptance of at-birth POC among parents of HIV-exposed infants. Furthermore, without being addressed, challenges with resources (primarily provider expertise and time), lack of provider experience with POC, and a misunderstanding of the mechanism of testing could limit clinicians’ ability and/or willingness to provide at-birth POC testing. Training for providers and counseling of parents will maximize the acceptance of at-birth POC testing among providers.

## Supporting information

S1 Checklist COREQ checklist. (PDF)

S1 File Parent interview guide. (DOCX)

S2 File Provider interview guide. (DOCX)

S3 File Parent code tree. (DOCX)

S4 File Provider code tree. (DOCX)
