# Supplementary material for: Factors affecting acceptance of at-birth point of care HIV testing among providers and parents in Kenya: A qualitative study
Source: PLoS One. 2019 Nov 22;14(11):e0225642. doi: 10.1371/journal.pone.0225642 (PMC6874324; doi:10.1371/journal.pone.0225642)
Supplement: S1 File — (DOCX) [file pone.0225642.s002.docx]

**KEY INFORMANT INTERVIEW GUIDE: HIV+ MOTHERS (HOSPITAL DELIVERY)/PARTNERS**

**STUDY TITLE:**

**Piloting at-birth point of care HIV testing strategies in Kenya**

**Introductory/Overview Questions**

1. Could you tell me a bit about your experience with EID services?
   1. What are some of the challenges you faced in receiving this care?
   2. What are some of the things that helped you receive this care?
   3. What are some things that would have helped you receive this care?
   4. How old was your infant when you enrolled in EID services?
2. Can you describe the process from when your infant was tested to when you received the results?
   1. How long did this process take?
   2. How did you feel while you were waiting to hear your infant’s test results?
   3. How did you feel about the length of time it took to receive the results?
3. Where did you deliver your baby?
   1. Who was with you during delivery?

*Probe: Health care provider? Midwife? Traditional birth attendant? Family/friends?*

1. Can you please describe the various services you and your infant received at the time of delivery and afterwards?
   1. Describe the care received for your infant.

*Probe: Tests, Checkups, Counseling, etc?*

- 1. Describe the care you received yourself.

P*robe: Tests, Checkups, Counseling, etc?*

1. Can you please describe the various services you and your infant accessed in the first 6 weeks after delivery?

**Questions About At-Birth Testing**

1. How do you feel about the idea of testing infants for HIV at birth?
   1. What do you think are some of the potential benefits of at birth testing?
   2. What do you think are some of the potential risks of testing infants at birth?
   3. Are there any other concerns you think mothers would have about testing their infant at birth?
2. Thinking about the process of delivery, can you think of any ways at birth testing could create a risk of disclosure?
   1. If an infant was tested for HIV at birth, who may find out about the mother’s status without her consent?

*Probe: Her partner? A Friend or relative? Clients at the hospital? Service providers?*

- 1. What are some ways providers can protect the confidentiality of mothers and their infants when testing for HIV at birth?

1. Thinking about the services mothers receive during delivery and afterwards, what would be the best time during that process to be introduced to the EID process?
2. If mothers were going to have their infants tested before being discharged from the hospital, at what point during the process do you think the test should be done?
3. If an infant could be tested at birth, what information would be helpful for mothers to know beforehand?
   1. How can providers prepare mothers for at birth testing?
   2. What types of information would make mothers more likely to accept at birth testing?
4. What are some reasons that mothers would want their infants tested at birth?
5. What are some reasons that mothers would not want their infants tested at birth?

**Questions about Point-of-Care (POC) Tests**

1. How do you think same day infant test results will affect EID services?
2. How do you think mothers would feel about HIV tests that can give results within the same day?
   1. Thinking back to how you felt while waiting for your infant’s test results, how would you feel different while waiting for test results on the same day the test was done?
   2. How do you think same-day results will affect mothers’ EID experience? How would having results quickly make a difference?
   3. How will same-day results affect mothers’ willingness to participate in EID?
   4. What are some reasons that mothers would want their infant’s test results in the same day?
      *Probe: What are some potential benefits?*
   5. What are some reasons that mothers would not want their infant’s test results in the same day?
      *Probe: What are some potential risks?*
3. Would you want to know your infant’s HIV status before leaving the hospital? Why or Why not?
4. How can providers prepare mothers for any results that they might receive?
5. Are there any other concerns or questions you have about point-of-care tests with same-day results?

**Questions about Community Based Testing**

1. In your opinion, what would be some benefits of using POC systems for HIV testing in the community?
2. From your perspective, what would be some challenges associated with providing community-based HIV screening using POC systems?
   1. What concerns related with unintentional disclosure of HIV status do you think mothers would have related to community-based HIV testing for infants?
   2. What concerns would you have regarding counselling, treatment, and follow up for mothers of positive infants?
3. What are some ways that each of these concerns and challenges can be minimized?
4. If point of care EID testing was available in your community, do you think mothers would access it for their infants? Why or why not?

*Probe: What level of demand would you anticipate?*
