# Supplementary material for: Factors affecting acceptance of at-birth point of care HIV testing among providers and parents in Kenya: A qualitative study
Source: PLoS One. 2019 Nov 22;14(11):e0225642. doi: 10.1371/journal.pone.0225642 (PMC6874324; doi:10.1371/journal.pone.0225642)
Supplement: S2 File — (DOCX) [file pone.0225642.s003.docx]

**KEY INFORMANT INTERVIEW GUIDE: PROVIDERS**

**STUDY TITLE:**

**Piloting at-birth point of care HIV testing strategies in Kenya**

**Introductory/Overview Question**s

1. Could you tell me a bit about your roles and responsibilities here at the hospital?
   1. How are you involved in maternity, PMTCT, and/or EID services?
   2. What are some of the challenges that you have experienced in providing these services?
2. Can you please walk me through the services that a laboring woman and her newborn receive, from the time the mother delivers to when they are discharged?
   *Probe: What department is she registered in? After delivery, which department(s) does she go to? How long will she and her infant stay in the hospital for SVD and for C-Section? What tests, checkups, etc. do mom and baby receive before being discharged?*

**Questions about At-Birth Testing**

1. From your perspective, what is the benefit of providing at-birth testing for HIV in infants?
   *Probe: How will it affect infant age at diagnosis? Effect on age at ART initiation?*
   *Probe: How will at birth testing affect the number of follow up appointments?*

*Probe: How will at birth testing affect time to notification of results?*
*Probe: Do you think it will affect loss to follow up? In what ways?*

1. What are some barriers you anticipate to providing at-birth testing for HIV-exposed infants?
   1. Do you foresee any barriers for the health care facility?
      *Probe: Feasibility of collecting a sample before a new mother is discharged?*
   2. Do you foresee any barriers for central laboratories?
      *Probe: Increased workload? Impact on turnaround time?*
2. How do you think collecting an infant sample before the mother leaves labor and delivery might pose any concerns for disclosure or stigma? In what ways?
   1. How can these concerns be reduced?
3. How do you think at-birth testing can be incorporated into the current workflow?
   1. Where will the infants be tested (L & D, Maternity, MCH)?
   2. Who will obtain the infant DBS for at-birth testing?
   3. Given the services provided to newborns that you described earlier, when would be the best time to do the newborn’s HIV test before discharge?
   4. What additional resources or support would be needed in order to implement at-birth testing for HIV-exposed infants?
4. Are there any specific concerns related to sample collection for infants born at certain times (i.e. during the week, after normal hospital hours, or on weekends)?

**Questions about Point-of-Care (POC) Tests**

1. What do you think are some benefits of using POC tests like Alere-Q and GeneXpert for early infant diagnosis for mothers and caregivers?
2. What are some reasons that mothers and caregivers would prefer to receive same-day results for their infant’s HIV test?
3. What are some reasons that mothers and caregivers would not prefer same day results?
4. What would be the benefit of same-day EID results to providers, if any?
5. Would same day EID results pose any challenges to providers?

*Probe: Time constraints? Resource constraints? Linkage to care?*

1. What are some concerns providers might have about using these new POC systems for routine EID services?

*Probe: Mother acceptance, provider workload, flow of admitted mothers at the hospital, provider training, Cartridge/computer/machine storage, maintenance issues?*

1. How could these provider challenges and concerns be minimized?

**Questions about incorporating POC tests into current workflow**

1. Who would be responsible for performing at-birth POC tests? POC tests at 6 weeks?
   1. How would performing these tests impact this person’s ability to complete their other responsibilities?
   2. How would this impact patient care?
2. How do you think routine POC testing can be incorporated into the current workflow?
   1. Where will the infants be tested? At birth? At 6 weeks?
   2. What additional resources or support would be needed in order to implement point of care HIV testing for HIV-exposed infants?
   3. What implications would incorporating POC tests into current workflow have for staff? For patients?
3. Do you have any concerns regarding secure equipment storage that need to be addressed? Where do you foresee any POC systems being stored?
4. How do you foresee POC results being used by hospital staff?
   1. When would caregiver notification occur?
   2. Given a positive POC result, what would be the timeline for infant ART initiation?
      *Probe: would anything need to occur before an infant was started on treatment? if so, what and when would this occur?*
   3. How confident would providers feel initiating ART based on positive point of care testing results?
      *Probe: What role will confirmatory PCR testing play alongside POC testing, if any?*
   4. How comfortable would you feel starting a 1-2 day old infant with a confirmed HIV-positive diagnosis on ART.
      *Probe: What factors would make you more comfortable doing so. What factors make you less comfortable doing so?*
5. Do you have any advice or suggestions regarding at-birth HIV testing or the use of POC tests for EID that you would like to share?
