# Supplementary material for: Factors affecting acceptance of at-birth point of care HIV testing among providers and parents in Kenya: A qualitative study
Source: PLoS One. 2019 Nov 22;14(11):e0225642. doi: 10.1371/journal.pone.0225642 (PMC6874324; doi:10.1371/journal.pone.0225642)
Supplement: S3 File — (DOCX) [file pone.0225642.s004.docx]

Parent Code Tree

[Environmental/Organizational Context 2](#_Toc19702162)

[Maternal burden 2](#_Toc19702163)

[Turnaround time of results 2](#_Toc19702164)

[SOC delivery/EID services 2](#_Toc19702165)

[Best Available Evidence 2](#_Toc19702166)

[Sensitivity/specificity 2](#_Toc19702167)

[Patient factors 2](#_Toc19702168)

[Patient characteristics 2](#_Toc19702169)

[Delivery location 2](#_Toc19702170)

[Disclosure status 2](#_Toc19702171)

[Employment 2](#_Toc19702172)

[Experience with EID 2](#_Toc19702173)

[Infant age 2](#_Toc19702174)

[Marital status 2](#_Toc19702175)

[Parity 2](#_Toc19702176)

[Experiences of stigma 2](#_Toc19702177)

[Timing of HIV diagnosis 2](#_Toc19702178)

[Patient needs 2](#_Toc19702179)

[Social support 2](#_Toc19702180)

[Confidentiality 2](#_Toc19702181)

[Counseling 2](#_Toc19702182)

[Patient preferences 2](#_Toc19702183)

[Turnaround time of results 2](#_Toc19702184)

[Timing of testing 2](#_Toc19702185)

[Timing of results 2](#_Toc19702186)

[Barriers to testing 2](#_Toc19702187)

# Environmental/Organizational Context

## Maternal burden

## Turnaround time of results

## SOC delivery/EID services

# Best Available Evidence

## Sensitivity/specificity

# Patient factors

## Patient characteristics

### Delivery location

### Disclosure status

### Employment

### Experience with EID

### Infant age

### Marital status

### Parity

### Experiences of stigma

### Timing of HIV diagnosis

## Patient needs

### Social support

### Confidentiality

### Counseling

## Patient preferences

### Turnaround time of results

### Timing of testing

### Timing of results

#### Anxiety

#### Infant care

### Barriers to testing

#### Fear/anxiety

#### Ignorance

#### Infant care

#### Infant comfort

#### Stigma/disclosure
