# Supplementary material for: Factors affecting acceptance of at-birth point of care HIV testing among providers and parents in Kenya: A qualitative study
Source: PLoS One. 2019 Nov 22;14(11):e0225642. doi: 10.1371/journal.pone.0225642 (PMC6874324; doi:10.1371/journal.pone.0225642)
Supplement: S4 File — (DOCX) [file pone.0225642.s005.docx]

Provider Code Tree

[Provider preferences 1](#_Toc19702340)

[Timing of results 1](#_Toc19702341)

[Inform infant care 1](#_Toc19702342)

[LTFU 1](#_Toc19702343)

[Workload 1](#_Toc19702344)

[Disclosure 1](#_Toc19702345)

[Patient needs 1](#_Toc19702346)

[Confidentiality 1](#_Toc19702347)

[Counseling 1](#_Toc19702348)

[Retention 1](#_Toc19702349)

[Patient preferences 1](#_Toc19702350)

[Timing of results 1](#_Toc19702351)

[anxiety 1](#_Toc19702352)

[stigma/disclosure 1](#_Toc19702353)

[Patient barriers 1](#_Toc19702354)

[Stigma/disclosure 1](#_Toc19702355)

[Infant comfort 1](#_Toc19702356)

[Infant care after diagnosis 1](#_Toc19702357)

[Fear/anxiety 1](#_Toc19702358)

[Resources 1](#_Toc19702359)

[Expertise 1](#_Toc19702360)

[ART initiation 1](#_Toc19702361)

[Mentor mothers 1](#_Toc19702362)

[Electricity 1](#_Toc19702363)

[Shortage of staff 1](#_Toc19702364)

[Space 1](#_Toc19702365)

[Availability of supplies 1](#_Toc19702366)

[Best available evidence 1](#_Toc19702367)

[Presence of antibodies 1](#_Toc19702368)

[Quality of machines 1](#_Toc19702369)

[Patient characteristics 2](#_Toc19702370)

[Timing of HIV diagnosis 2](#_Toc19702371)

[Environmental Context 2](#_Toc19702372)

[SOC services 2](#_Toc19702373)

[Patient barriers 2](#_Toc19702374)
